# A configurational pathway study on enhancing the governance level of national fitness public services

**DOI:** 10.3389/fpubh.2026.1860547

**Published:** 2026-09-10

**Authors:** Xiaoxiu Song, Qun Qiao

**Affiliations:** 1 School of Public Administration, China University of Geosciences (Wuhan), Wuhan, Hubei, China; 2 School of Sports Training, Wuhan Sports University, Wuhan, Hubei, China

**Keywords:** configurational analysis, governance efficiency, multi-path analysis, national fitness, public service governance

## Abstract

**Objective:**

Relying solely on single-dimension explanations for differences in the governance performance of national fitness public services makes it difficult to reveal the complex mechanisms of multi-element synergistic interaction. This study aims to break through this limitation by adopting a system perspective to deeply explore the complex causal mechanisms driving the enhancement of national fitness public service governance efficacy.

**Methods:**

(1) Introducing the “Wuli-Shili-Renli” (WSR) system methodology to build an analytical framework encompassing three dimensions: resource foundation, institutional mechanisms, and governance actors' capabilities; (2) Applying the fuzzy-set Qualitative Comparative Analysis (fsQCA) method; (3) Conducting a configuration path analysis using 31 provincial-level governments in China as cases.

**Results:**

(1) High governance efficacy does not result from any single necessary condition, but rather from the concurrent interactions of multi-dimensional elements; (2) Three equivalent paths can achieve high governance efficacy: “Resource-Driven,” “Institution-Driven,” and “Collaborative Governance”; (3) Path selection displays significant regional heterogeneity, exhibiting a complementary relationship of “common patterns with situational differences.”

**Conclusion:**

From a system perspective, this study deepens the understanding of the complex causal mechanisms behind national fitness public service governance. It offers theoretical references and path guidance for enhancing governance efficacy-namely, that governance efficacy improvement should be grounded in the synergistic configuration of multiple elements and tailored to regional realities through differentiated implementation paths, rather than relying on linear input along a single dimension.

## 1 Introduction

### 1.1 Research significance

In recent years, the construction of China's national fitness public service system has deepened continuously under the impetus of national strategies. Its governance process encompasses multiple dimensions, including resource supply, institutional operation, actor coordination, and policy implementation, exhibiting an increasingly diversified, collaborative, and refined development trajectory (1). With the rapid growth of public demand for high-quality fitness services, public service governance has expanded beyond traditional venue and facility construction toward institutional improvement, social collaboration, digital empowerment, and capacity enhancement. In this process, the governance role of local governments has shifted from that of a “mere provider” to that of an “institutional guide, coordinator, and regulator,” and the governance logic has evolved from “resource-input-driven” to “resource-institution-actor synergy-driven,” with governance practices exhibiting increasingly complex systemic characteristics. Governance theory research indicates that, in complex public service systems, different factors often interact through various combinatory patterns and may form multiple functionally equivalent realization pathways (2). Therefore, neglecting the combinatory relationships among factors risks underestimating the critical role of institutional arrangements and governance capacity in shaping public service performance.

From the perspective of practical operations, under a unified national policy framework, significant disparities in national fitness public service performance persist across different regions—disparities that cannot be fully explained by economic development levels or the scale of fiscal investment alone. Public governance research widely suggests that when a public service system enters a relatively mature stage, governance performance depends more on structural factors such as institutional arrangements, organizational coordination, and execution capacity, rather than on the simple aggregation of individual input factors (3). Accordingly, a pressing theoretical question emerges: Through which combinatory configurations of factors is high governance effectiveness in national fitness public services actually achieved? And how do different pathways function under varying contexts? Answering these questions requires moving beyond the traditional linear “input-output” logic and examining the internal mechanisms of multi-factor synergistic interactions from a systemic structure and configurational perspective.

### 1.2 Literature review

Scholarship on national fitness public service governance, both domestically and internationally, has evolved into a relatively systematic body of research, with its foci shifting from an emphasis on material resource foundations, to attention to institutional supply and coordination mechanisms, and further to in-depth analyses of governance actor behavior and capacity. This evolution aligns closely, in logical terms, with the three-dimensional WSR framework adopted in this study.

(1) Wuli dimension: research on resource base and infrastructure provision
  Early studies largely treated national fitness public services as a classic public good, focusing primarily on the influence of fiscal investment, sports venues and facilities, and human resource allocation—the “Wuli” factors—on public service supply levels. Sallis et al. (4) noted that the density of public sports facilities was significantly and positively correlated with residents' physical activity levels. Shen and Lu (5), through an empirical analysis of provincial-level sports public service data in China, found that the intensity of fiscal sports expenditure and per capita sports venue area had significant positive effects on the level of national fitness public services. Li et al. (6), using Guangdong Province as a case study, found that the level of economic development, population density, and fiscal investment were important factors influencing the Wuli supply efficiency of national fitness public services. However, as practice has deepened, Liu et al. (7) noted that phenomena such as sports facilities being “built but unused” and “managed separately from use” revealed a structural tension between Wuli factor supply and governance mechanisms. While the above studies have illuminated the foundational role of Wuli factors from a resource allocation perspective, their common limitation lies in treating resource inputs as sufficient conditions for governance performance, failing to explain the co-occurrence of the empirical realities of “high input, low performance” and “low input, high performance.”
(2) Shili dimension: research on institutional design and operational mechanisms

With the basic formation of the national fitness public service system framework, the research focus shifted significantly to the Shili level, encompassing institutional design, policy instruments, and coordination mechanisms. Shi and Dai (8) constructed a performance evaluation indicator system for national fitness public services from the perspective of government procurement of services, pointing out that institutional design, and operational mechanisms play a critical role in transforming service performance. Wang et al. (9) systematically reviewed the process of policy system construction, arguing that the continuous improvement of the policy system constitutes an important prerequisite for enhancing public service quality. Chen and Chen (10) noted that building a higher-level national fitness public service system requires properly handling the relationship between government leadership and social force participation. Xue et al. (11), through fsQCA analysis, found that authoritative instruments tended to exhibit higher effectiveness within configurations, whereas capacity-building instruments, though supplied in greater numbers, often appeared as peripheral conditions. Yuan and Chen (12), based on urban public sports facility management practices, proposed a “cohesive governance model,” emphasizing the coordination, integration, and institutional embedding functions of government in multi-actor interactions. Ru et al.'s (13) empirical research demonstrated that national fitness basic public service standards, during local implementation, produce diverse implementation patterns owing to variations in factors such as resource conditions and attention allocation. These studies have profoundly revealed the pivotal role of institutional mechanisms in public sports service governance, yet most research remains focused on the degree of institutional completeness per se, paying insufficient attention to how institutions interact synergistically with resources, capacity, and other factors.

(3) Renli dimension: research on actor capabilities and behavioral strategies
  In recent years, concomitant with the broader paradigm shift in public governance, the research frontier has increasingly focused on the Renli level—encompassing government execution capacity, social organization capability, and public participation—emphasizing that the agency of governance actors is central to determining governance outcomes. Scholarship in this vein contends that ultimate governance performance depends not only on static resources and institutions, but more critically on the interactive relationships, behavioral strategies, and capability levels of various actors. Zhu and Cao (14), through evolutionary game model analysis, pointed out that enhancing the initial willingness for collaboration among all parties can effectively drive the stable realization of multi-actor collaborative supply of national fitness public services. Zhou et al. (15) argued that social organizations, through institutional embedding, resource integration, and specialized services, help compensate for the boundaries of government governance, and that their organizational capacity and depth of embeddedness are important factors in enhancing the governance effectiveness of sports public services. Ma et al. (16) noted that interregional disparities in governance actor capabilities and behavioral styles constitute a significant factor contributing to uneven resource allocation efficiency across regions. The above studies have shifted the analytical focus to the agency of “people,” but most remain confined to single-dimensional investigations, lacking a systematic explanation of how the three categories of factors—Wuli, Shili, and Renli—jointly determine governance performance through their combinatory configurations.

### 1.3 Research gaps

Synthesizing the above literature, the following three notable inadequacies exist in existing research:

First, at the level of theoretical frameworks, most studies are confined to single-indicator or single-dimensional analyses, lacking systematic framework depictions of the complexity of public service governance. In the empirical world, Wuli, Shili, and Renli factors do not operate independently but appear in combinatory forms; yet existing research lacks an analytical framework capable of integrating these three categories of factors, making it difficult to reveal the internal logic of their synergistic configuration.

Second, at the methodological level, existing empirical studies are dominated by traditional regression analysis, which focuses on the “net effect” of individual variables and is poorly equipped to answer the critical question of “how multiple factors combine to produce high governance effectiveness.” The phenomenon of equifinality that is prevalent in reality—wherein different factor combinations may yield the same governance outcome—can only be effectively identified through configurational analysis methods.

Third, at the level of practical explanation, existing research struggles to account for paradoxical phenomena such as “abundant resources yet low governance effectiveness” or “limited resources yet outstanding governance performance,” and there is a shortage of inquiry into how governance factors form multiple effective pathways through different combinations.

Based on the above research gaps, this paper constructs an integrative analytical framework grounded in the WSR system methodology and employs fuzzy-set qualitative comparative analysis to systematically identify the multiple conditional configurations for enhancing the governance effectiveness of national fitness public services, with the aim of revealing the internal mechanism of “multi-factor configuration—multi-pathway generation.”

## 2 Theoretical foundation

To analyze in depth the complex causal mechanisms underlying the enhancement of governance effectiveness in national fitness public services, this study adopts the Wuli-Shili-Renli (WSR) system methodology as its core theoretical framework. WSR is a methodology characterized by Eastern systems philosophy; it emphasizes that any effective intervention in and management of a complex socio-technical system must comprehensively consider three dimensions: objective material conditions (Wuli), the operational logic of the system (Shili), and the interpersonal relationships and value judgments of relevant stakeholders (Renli) (17). WSR theory posits that Wuli factors provide the foundational conditions of the governance system, Shili factors embody the institutions and methods of the governance process, and Renli factors reflect the capabilities, motivations, and relationships of governance actors. The three dimensions do not follow a simple linear causality; rather, they interact, function jointly, and reinforce one another. Accordingly, high-level governance of public services does not depend on any single factor but results from the combined action of multiple factors. This theoretical perspective aligns closely with the practical characteristics of national fitness public service governance, because the national fitness system involves multiple categories of governance factors—including venues and facilities, funding investment, institutional systems, cross-departmental coordination, organizational participation, and public behavior—among which relationships are often not unidirectional but exhibit combinatory interactions. The applied value of the WSR methodology in public service research is precisely reflected in its capacity to integrate individual behavior, organizational interaction, and the institutional environment into a unified analytical framework, thereby avoiding the research bias of “emphasizing institutions while neglecting behavior.” This viewpoint provides an important methodological basis for introducing WSR into research on national fitness public service provision.

Based on WSR theory, the governance factors of national fitness public services can be systematically depicted along the three dimensions of Wuli, Shili, and Renli. The Wuli dimension primarily embodies the material foundation of public services, including the level of fiscal investment, the quantity and accessibility of public sports venues, the supply of sports professionals, and the construction of digital platforms, addressing the question of “whether governance has the necessary resources.” The Shili dimension embodies governance mechanisms and institutional rules, including the completeness and enforcement strength of the policy system, coordination mechanisms within and across government departments, social organization participation mechanisms, and the quality monitoring and evaluation systems for public services, reflecting “whether governance is scientific, efficient, and orderly.” The Renli dimension focuses on governance actor behavior and capacity, including government execution capacity, grassroots organizational mobilization capacity, the activity level and professional standards of social sports organizations, and residents' sports participation behavior and cultural atmosphere, addressing the question of “who conducts governance and how well it is done.”

From the perspective of system logic, the three dimensions are characterized by holism, synergy, and complementarity. The Wuli dimension provides the foundational support for public services, but whether resources can be transformed into effective services depends on the institutional mechanisms of the Shili dimension, and the operational effectiveness of these institutional mechanisms in turn depends on the capabilities and behaviors of governance actors within the Renli dimension. For example, regions with abundant resources but inefficient institutions and insufficient execution capacity may experience “resource waste,” whereas regions with limited resources but sound institutions and proactive actors can often achieve higher levels of public services through “institutional compensation.” In other words, high-level governance of national fitness public services is not driven by a single factor but presents multiple effective pathways under different developmental stages, in different regions, and under different governance conditions.

On this basis, this paper constructs an analytical framework for national fitness public service governance suitable for empirical research. This framework adopts WSR as its structural foundation, classifying governance conditions into the three broad categories of Wuli, Shili, and Renli, and defining the governance outcome as a composite index of “the governance level of national fitness public services.” The Wuli dimension mainly encompasses fiscal resources, facility resources, and human resources; the Shili dimension primarily includes the degree of policy system completeness, cross-departmental coordination mechanisms, and social participation mechanisms; the Renli dimension comprises government execution capacity, social organization activity levels, and residents' sports participation behavior. These three dimensions jointly constitute the systemic structure of national fitness public service governance and determine the ultimate expression of governance level.

## 3 Research design

### 3.1 Methodological selection: applicability of QCA

In the governance of national fitness public services, a quintessentially complex public governance issue, the formation of governance effectiveness does not stem from the linear driving force of a single factor but rather from the interaction and synergistic configuration of multidimensional conditions in specific contexts. On this basis, this study introduces qualitative comparative analysis (QCA) in its methodological selection to identify the multiple pathways through which different factor combinations drive the improvement of governance effectiveness.

Qualitative comparative analysis (QCA) is a case-oriented research method grounded in set theory and Boolean algebra. The method seeks to integrate the depth of qualitative research with the breadth of quantitative research, and it is particularly suited to addressing complex causal problems with small to medium sample sizes. Unlike traditional regression analysis, which focuses on the net effects of individual variables, the core strength of QCA lies in its ability to identify multiple combinations of conditions that lead to the same outcome. From a theoretical logic perspective, the WSR systems methodology adopted in this paper exhibits a high degree of internal consistency with the QCA method in terms of their complex causal explanatory frameworks. WSR emphasizes that public service governance performance arises from the synergistic interaction among Wuli, Shili, and Renli elements. No simple linear causal relationship exists among these three categories of elements; rather, they jointly act upon governance outcomes through multiple combinations, exhibiting typical systemic and configurational characteristics. This, in essence, is a complex causal perspective in which multiple conditions combine to produce outcomes. Correspondingly, the QCA method, grounded in set theory, emphasizes conjunctural causation, equifinality, and causal asymmetry, and is capable of answering the question: “Under what combinations of conditions does the outcome occur?” Compared with traditional regression analysis, which emphasizes the independent net effects of variables, QCA pays greater attention to the interactive relationships among conditions and their configurational structures, and is able to identify multiple equifinal pathways leading to the same outcome. This methodological feature is highly compatible with the theoretical core of WSR, which emphasizes multi-element synergy and holds that different pathways can all achieve governance objectives.

The WSR methodology provides a systematic theoretical framework for analyzing the governance of national fitness public services, while the QCA method offers an effective analytical tool for identifying multi-element combination pathways. The inherent consistency between the two in complex causal explanatory logic allows the following analytical logic to be proposed: first, governance outcomes derive from the combination of multiple elements rather than the action of any single element; second, different elements exhibit both substitutability and complementarity, such that different combinations may produce the same level of governance; third, governance pathways are diverse, and various regions may achieve improved governance performance through different element combinations. This logic closely aligns with the fundamental concepts in qualitative comparative analysis, such as “multiple conjunctural causation,” “combinatorial effects,” and “equifinal pathways.” Thus, the WSR framework not only provides theoretical support for variable construction and indicator classification in this paper, but also lays the foundation for the subsequent configurational analysis.

### 3.2 Case selection and variable system construction

#### 3.2.1 Case selection

This study takes China's 31 provinces, autonomous regions, and municipalities directly under the central government (excluding Hong Kong, Macao, and Taiwan) in 2023 as the analytical cases. The choice of the provincial level as the unit of analysis was based on two considerations. First, the alignment between the level of analysis and governance actors. Within China's multi-level governance structure of “central–provincial–municipal–county–township,” provincial-level governments assume multiple functions, including refining and implementing national policies, coordinating the allocation of regional resources, and supervising and guiding municipal and county-level implementation. Their governance behaviors directly determine the supply level, resource allocation efficiency, and institutional implementation effectiveness of national fitness public services within their jurisdictions. Second, the balance between case homogeneity and heterogeneity. The QCA method requires that the selected cases possess a certain degree of comparability in key characteristics while also exhibiting heterogeneity in antecedent conditions and outcome variables. In terms of homogeneity, all 31 provinces operate under a unified central policy framework, follow the same national fitness development plan and assessment requirements, and therefore possess a fundamental basis for comparability. In terms of heterogeneity, the provinces exhibit significant differences in economic development levels, resource endowments, institutional environments, and governance capacities; such differences provide the necessary case variation space for this study to identify diverse configurational pathways. A sample size of 31 cases falls within the applicable range of the QCA method, as it is sufficiently large to ensure case heterogeneity for revealing diverse pathways while avoiding the pitfalls of traditional large-sample statistical paradigms.

#### 3.2.2 Variable system: construction based on the WSR framework

Drawing on the WSR systems methodology, this study constructed an analytical system comprising one outcome variable and nine conditional variables, with all variables measured through observable objective indicators.

(1) Outcome variable: government governance effectiveness of national fitness public services

From the perspective of theoretical connotation, government sports public service governance effectiveness is not a unidimensional measure of performance output or service scale; rather, it is a comprehensive reflection of multidimensional governance capacity and governance outcomes. Compared with traditional approaches that measure public service levels using single indicators, the governance effectiveness of national fitness public services places greater emphasis on the comprehensive expression of multidimensional performance outcomes, bearing distinct comprehensive, and outcome-oriented characteristics. Following the approach of Wang (18), and integrating the policy attributes and governance practices of national fitness public services, this paper contextualizes and operationalizes the concept of governance effectiveness, adopting “governance effectiveness of national fitness public services” as the outcome variable in the fsQCA analysis. It comprehensively captures the integrated capacity and actual performance of provincial-level governments in transforming governance inputs into public service outcomes through resource allocation, institutional operation, and actor coordination in the domain of national fitness public services (18). This paper systematically reviewed the China Sports Statistical Yearbook, the China Statistical Yearbook, the China Health Statistics Yearbook, and the publicly available annual reports of provincial-level sports administrative departments and health commissions, constructing a comprehensive evaluation system based on service accessibility, service satisfaction, and health outcomes. Given the different dimensions of the three indicators and to avoid the influence of extreme values, min-max normalization was employed to eliminate dimensional differences. A weighted sum method was used to calculate the comprehensive governance effectiveness index for each province, which served as the qualitative standard for QCA analysis, enabling an objective mapping of the relative rankings of different provinces on the frontier of national fitness public service governance. The calculation formulas are as follows.


$$\begin{array}{l} x^{*}=\frac{x-x_{\min}}{x_{\max}-x_{\min}} \end{array}$$ (1)

where xmin and xmax are the minimum and maximum values of the indicator across the 31 provinces, respectively, and x^*^ is the standardized indicator value.


$$\begin{array}{l} e_{\text{j}}=\;-\frac{1}{\text{lnn}}\overset{\text{n}}{\underset{\text{i}=1}{\sum }}{\text{p}}_{\text{ij}}\text{ ln}{\text{p}}_{\text{ij}},\;{\text{p}}_{\text{ij}}=\frac{{\text{X}}_{\text{ij}}^{\text{′}}}{\sum_{\text{i}=1}^{\text{n}}X_{\text{ij}}^{\prime }} \end{array}$$ (2)

where n is the total number of provinces, j represents the index number of each evaluation indicator; Xij is the standardized value of the i-th province on the j-th indicator calculated using Equation 1; pij represents the proportion of the i-th province's value on the j-th indicator relative to the sum of that indicator across all provinces.


$$\begin{array}{l} {\text{d}}_{\text{j}}=1-{\text{e}}_{\text{j}},\;{\text{w}}_{\text{j}}=\frac{{\text{d}}_{\text{j}}}{\sum_{\text{j}=1}^{3}{\text{d}}_{\text{j}}} \end{array}$$ (3)

where m is the total number of indicators, dj is the information redundancy (coefficient of variation) of the j-th indicator, and wj is the entropy weight of the j-th indicator. On this basis, the weighted-sum method was used to calculate the comprehensive governance effectiveness index of national fitness public services for each province, as shown in Equation 4.


$$\begin{array}{l} \text{G}{\text{E}}_{\text{i}}=\overset{3}{\underset{\text{j}=1}{\sum }}{\text{w}}_{\text{j}}\times {\text{X}}_{\text{ij}}^{\text{′}} \end{array}$$ (4)

where GEi denotes the comprehensive governance effectiveness index of national fitness public services for the i-th province.

Raw data sources and distribution characteristics of the three-dimensional sub-indicator:

The specific composition and data sources of the three sub-indicators comprising the Governance Efficacy Composite Index (GE) are as follows: (1) Service Accessibility: This dimension is measured using two indicators: “per capita sports venue area (square meters per capita)” and “proportion of townships (sub-districts) covered by public sports venues (%).” Data are derived from the 2023 Sports Industry Statistical Yearbook and annual reports published by provincial sports bureaus. (2) Service Satisfaction: This dimension is measured by the 'resident satisfaction with public sports services' score (out of 100) as reported in the National Fitness Status Survey Reports of each province. Data are obtained from the provincial survey data publicly released by the Mass Sports Department of the General Administration of Sport of China. (3) Health Outcomes: This dimension is measured using two indicators: “national physical fitness compliance rate among urban and rural residents (%)” and “proportion of the population regularly participating in physical exercise (%).” Data are sourced from the 2023 China Health Statistical Yearbook and provincial physical fitness monitoring bulletins.

The above three dimensions correspond respectively to the full chain of public service: “supply coverage–user evaluation–policy output.” They are both theoretically grounded and aligned with the policy objective system for national fitness public services. The weight allocation employed the entropy weight method (see Equations 2, 3), which determines the objective weights of each dimension in a data-driven manner, thereby avoiding the bias introduced by subjective weighting. The calculated weights were: service accessibility (w1 = 0.342), service satisfaction (w2 = 0.315), and health outcomes (w3 = 0.343). The balanced distribution of the three weights indicates that all three indicators contribute approximately equal explanatory power to the comprehensive evaluation of governance effectiveness.

In addition, to verify the robustness of the composite index construction, this study conducted a sensitivity test using the equal-weight method (i.e., each dimension assigned a weight of 1/3). The results showed that the Spearman correlation coefficient of provincial governance effectiveness rankings under the equal-weight method reached above 0.95, demonstrating a high degree of consistency with the entropy weight method results. This indicates that the composite index is insensitive to weight settings and that the conclusions are robust.

(2) Conditional variables: nine antecedents across the three WSR dimensions

The construction of the conditional variables strictly followed the three-dimensional logic of “Wuli-Shili-Renli” in the WSR systems methodology. Nine representative antecedent conditions were selected around the core elements of national fitness public service governance, ensuring precise alignment between the variables and the theoretical dimensions.

The selection of the above nine conditional variables (Table 1) followed a rigorous dual “theory–experience” logic:

**Table 1:** Conditional variable definitions.

Dimension	Variable name	Symbol	Operational definition and measurement
Wuli	Facility level	SC	Number of national fitness projects
Wuli	Fiscal support intensity	FE	Per capita mass sports expenditure
Wuli	Social organization scale	SO	Per capita number of sports social organizations
Shili	Institutional completeness	AA	Number of sports venue institutions
Shili	Policy supply intensity	PP	Annual average number of special national fitness policies
Shili	Activity frequency	HE	Number of public health education activities
Renli	Government service capacity	GC	Provincial government integrated service capacity assessment score
Renli	Social sports instruction	SI	Number of social sports instructors
Renli	System human resources	SE	Total number of sports system employees

From the Wuli dimension, facility level (number of national fitness projects) represents the material carrier of public services and serves as the spatial foundation for residents' fitness activities; fiscal support intensity (per capita mass sports expenditure) represents the financial lifeblood of public services, determining the sustainability of facility construction and operational maintenance; and social organization scale (per capita number of sports social organizations) represents the organizational skeleton of public services, reflecting the activeness of social forces participating in service provision. These three indicators correspond respectively to the triple resource foundations of “hardware–funding–organization,” jointly constituting a complete measurement of the Wuli dimension. This operationalization scheme draws on the established practices of studies such as Shen and Lü (5) and Wang (18).

From the Shili dimension, institutional completeness (number of sports venue institutions) reflects the degree of organizational structuring of the governance system and serves as the carrier guarantee for institutional operation; policy supply intensity (annual average number of special national fitness policies) reflects the level of institutionalization of governance, embodying policy continuity and systematicity; and activity frequency (number of public health education activities) reflects the routine operational capacity of governance, serving as a direct manifestation of institutions moving from text to practice. Together, these three indicators delineate the Shili logic of “whether institutions are complete and whether operations are orderly.”

From the Renli dimension, government service capacity (provincial-level government integrated service capacity assessment score) reflects the execution effectiveness of the government as the core governance actor; social sports instruction level (number of social sports instructors per 10,000 residents) reflects the allocation density of professional human capital; and system human resources (total number of sports system employees) reflects the scale foundation of the dedicated governance workforce. These three indicators delineate the full picture of actor capacity across three levels: “government execution capacity–professional service capacity–workforce support capacity.”

All nine indicators have been repeatedly validated in existing research as key variables influencing public service governance performance and exhibited sufficient discrimination in the exploratory data analysis of this study, satisfying the basic requirement of QCA for variability in conditional variables.

### 3.3 Data sources, processing, and calibration

#### 3.3.1 Data sources and preprocessing

The data for this study were primarily drawn from the 2023 China Sports Statistical Yearbook, the China Civil Affairs Statistical Yearbook, the statistical yearbooks of each province (autonomous region, municipality directly under the central government), and fiscal final account reports, covering key Wuli indicators and selected Shili and Renli indicators such as facility infrastructure, human resources, and fiscal expenditure. Policy-related data were obtained by retrieving provincial-level special national fitness policy documents published over the most recent 3 years from the PKULaw database. The retrieval and coding of policy texts followed these rules. Retrieval strategy: using “national fitness” and “sports public services” as keywords, normative documents issued by each province between January 1, 2021, and December 31, 2023, were searched in the PKULaw database. Inclusion criteria: (1) the issuing body was a provincial-level people's government or its constituent departments; (2) the document content was directly related to national fitness or public sports services; (3) the document type was a normative document such as “notice,” “opinion,” “plan,” or “measures,” excluding non-policy documents such as replies and official letters. Coding procedure: two researchers independently screened and coded the retrieval results, and a cross-check was conducted on 20% of the sample; the inter-coder reliability (Cohen's kappa) was 0.89, indicating high coding consistency. Policy supply intensity was ultimately measured as the annual average number of policy documents issued by each province. Government service capacity data were sourced from the Provincial Government Integrated Service Capacity Survey and Assessment Report published by the National Academy of Governance. Institutional completeness data were compiled through reviewing provincial and municipal government work reports and publicly available information from the official websites of sports departments. Public participation indicators were referenced from each province's National Fitness Status Survey Report. For the sporadic missing data in a few provinces, linear interpolation or reasonable estimation based on data trends from adjacent years was applied. Meanwhile, absolute value indicators such as fiscal expenditure and total number of employees were normalized on a per capita basis using year-end resident population figures, effectively eliminating scale effects and further enhancing cross-provincial data comparability, thereby laying a solid data foundation for the subsequent QCA analysis. For sporadic missing data in a few provinces, linear interpolation or reasonable estimation based on trends in adjacent years was applied. In terms of data missingness, the proportion of missing observations across all variables was less than 2%. Specifically, a few provinces (such as Tibet and Qinghai) had sporadic missing values for the indicators “number of national fitness projects” and “total sports system employees,” which were estimated using linear interpolation based on data trends from adjacent years (2022 and 2024). For “annual average special policies,” after verifying each province through the PKULaw database and provincial government websites, years without policy documents were assigned a value of 0. All absolute value indicators (fiscal expenditure, total number of employees, etc.) were normalized on a per capita basis using year-end resident population figures to eliminate scale effects and enhance cross-provincial comparability.

This study selected 2023 as the data time cross-section based on two considerations. First, 2023 was the mid-term assessment year for the implementation of the 14th Five-Year Plan for National Fitness, during which the completeness and comparability of statistical data from provincial-level governments were relatively favorable. Second, data from this year are able to reflect the normalized operational status of the recovery and development of national fitness public services in the post-pandemic transition period, carrying strong policy reference value. It should be noted that, due to the inherent time lag in the publication of statistical yearbooks and data compilation, the conclusions of this study primarily reflect the configurational relationships at the 2023 time cross-section; future research may test the stability of pathway structures using updated data.

#### 3.3.2 Descriptive statistical analysis

To comprehensively capture the basic distributional characteristics of the study variables, descriptive statistical analysis was conducted, and the results are presented in Table 2. Overall, a certain degree of dispersion was observed among the variables across different samples, indicating significant inter-regional differences in the governance of national fitness public services in China. The overall fluctuation in the government governance effectiveness index was relatively small, suggesting that all regions had attained a certain baseline level of national fitness public service governance, though differences still persisted. In contrast, resource-related variables such as the number of national fitness projects and the number of social sports instructors exhibited a high degree of dispersion, reflecting pronounced inter-regional imbalances in infrastructure and human resource allocation. With respect to per capita mass sports expenditure, the development of sports social organizations, and venue supply, significant inter-regional differences were likewise observed, reflecting imbalances in public service investment and the degree of social participation. Meanwhile, the supply of relevant policies and the implementation of health education activities also varied considerably, indicating regional differentiation in institutional supply and service outreach efforts. In summary, all variables exhibited varying degrees of dispersion, indicating that the governance of national fitness public services in China features certain differences across resources, institutions, and capacities, and that the sample in this study possesses adequate discrimination to meet the research requirements of the subsequent fuzzy-set qualitative comparative analysis.

**Table 2:** Descriptive statistics.

Variable	Max	Min	Std	Mean
National fitness projects	11,547.000	795.000	2,582.377	4,764.677
Per capita mass sports expenditure	4,352.000	18.000	1,209.402	1,199.194
Per capita sports social organizations	278.900	11.526	51.107	50.939
Sports venue institutions	3.738	0.102	0.610	0.590
Annual average special policies	47.000	0.000	12.711	16.387
Public health education activities	8.000	0.000	2.348	2.387
Provincial government service capacity	6,427.000	205.000	1,703.631	2,346.065
Social sports instructors per 10,000 residents	96.730	74.070	6.478	84.091
Sports system employees	540,666.000	15,331.000	128,855.029	173,913.839

#### 3.3.3 Fuzzy-set calibration

QCA analysis requires that variable values be transformed into set membership scores. Using the direct calibration method, the 75th percentile, the mean, and the 25th percentile of the sample were set as the thresholds for full membership, the crossover point, and full non-membership, respectively. Simultaneously, to avoid cases being excluded from analysis due to membership scores of exactly 0.5 at the crossover point, membership scores of 0.5 were manually adjusted to 0.501. The setting of calibration anchors in this study integrated the actual distribution of the sample data, national policy target values, and commonly used thresholds in relevant academic research to determine three theoretical or empirical anchors for each variable. The resulting anchors are presented in Table 3.

**Table 3:** Variable calibration results.

Variable		Variable type		
		Full non-membership (25%)	Crossover point (50%)	Full membership (75%)
Government governance effectiveness index	Outcome variable	0.725	0.757	0.78
National fitness projects	Conditional variable	366.5	1,199.194	1,648
Per capita mass sports expenditure	Conditional variable	25.824	46.344	50.939
Per capita sports social organizations	Conditional variable	0.353	0.59	0.61
Sports venue institutions	Conditional variable	6.5	16.387	22.5
Annual average special policies	Conditional variable	0	2.387	3.5
Public health education activities	Conditional variable	1,012.5	2,346.065	3,123
Provincial government service capacity	Conditional variable	79.665	84.091	89.21
Social sports instructors	Conditional variable	91,307	173,913.839	235,065
Sports system employees	Conditional variable	3,333	4,764.677	6,078

Qualitative Meaning of Calibration Anchors: In QCA, the setting of calibration anchors must be endowed with clear substantive meaning rather than relying solely on statistical percentiles. The qualitative meaning of each variable's calibration anchors in this study is as follows:

Full membership (75th percentile): indicates that the province has reached a “high level” of performance in a given condition dimension. For example, a value of national fitness projects greater than or equal to 1,648 indicates that the province already possesses sufficient foundational conditions in facility infrastructure provision to support a relatively high level of public service governance.

Crossover point (50th percentile, i.e., the median): indicates that the province is at a “moderate level” in that dimension, possessing neither a clear advantage nor a marked deficiency, and represents the fuzzy boundary distinguishing “high” from “low.”

Full non-membership (25th percentile): indicates that the province is at a “low level” in that dimension, with relatively scarce resources or capacity, making it difficult to sustain high governance effectiveness relying on that condition alone.

Taking “per capita mass sports expenditure” as an example: the full membership anchor is 50.939 yuan, indicating that when a province's per capita fiscal investment reaches this level, it can be regarded as having “sufficient fiscal support”; the full non-membership anchor ***is 25.824 yuan, indicating that below this level, fiscal*** ***support is clearly insufficient; and the crossover point of 46.344 yuan represents*** a moderate level. This calibration ensures that the resulting membership scores possess interpretable substantive meaning rather than merely reflecting statistical relative positions.

It should be noted that the “number of national fitness projects” exhibits a markedly right-skewed distribution across the 31 provinces: a small number of eastern provinces (e.g., Shandong with 11,547 projects, Guangdong, etc.) possess extremely high project counts, substantially inflating the overall mean, whereas the majority of provinces cluster at lower levels (the median is 1,199.194 projects). Accordingly, the calibration anchors are established based on the median (crossover point) and quartiles (full/non-membership thresholds), faithfully reflecting each province's relative position within the distribution rather than using the mean as a reference. This treatment aligns with the fundamental principle of QCA calibration—anchors should be based on the actual quantiles of the sample distribution, not on the mean or standard deviation.

## 4 Empirical analysis

### 4.1 Necessity analysis of individual antecedent conditions

Before conducting conditional configuration analysis, it is first necessary to test whether each individual conditional variable constitutes a “necessary condition” for the outcome. A necessary condition means that whenever the outcome occurs, the condition must also be present; if the condition is absent, the outcome cannot be produced. In QCA, the consistency indicator is typically used to measure the degree of necessity, and a condition is considered a necessary condition for the outcome when its consistency score exceeds 0.9.

This study conducted necessity tests for nine antecedent conditions and their negations, with results shown in Table 4. With the exception of “not-high per capita mass sports expenditure,” which had a consistency of 0.940, approaching the 0.9 threshold, all other conditional variables had consistency scores below 0.9. This result indicates that in the domain of government public service governance for national fitness, virtually no single condition can constitute a necessary condition for high governance effectiveness—even the “not-high per capita mass sports expenditure” condition that approached the threshold had a coverage of only 0.601, indicating that this condition explains only about 60% of high-effectiveness cases and fails to meet the universality standard of a strict necessary condition. Whether in terms of superior venue facilities, adequate fiscal guarantees, sound institutional arrangements, or strong government service capacity, no single-dimensional advantage alone is sufficient to ensure a high level of governance effectiveness. This finding reveals the complexity and systemic nature of government public service governance: the emergence of high effectiveness does not depend on any single factor but rather results from the synergistic, conjunctural effects of multiple factors. At the same time, the phenomenon of “not-high per capita mass sports expenditure” approaching the necessary condition threshold deserves attention, and its deeper implications will be further elucidated in Section 4.2 in conjunction with the configuration pathways. From the reverse perspective, the overall results of the necessity analysis confirm both the necessity and the appropriateness of adopting a configurational perspective to explore the conjunctural causality of multiple conditions.

**Table 4:** Results of necessity analysis of individual antecedent conditions.

Condition	High effectiveness		Non-high effectiveness	
	Consistency	Coverage	Consistency	Coverage
High SC	0.573	0.833	0.252	0.275
Not-high SC	0.501	0.472	0.846	0.598
High FE	0.121	0.657	0.166	0.676
Not-high FE	0.940	0.601	0.916	0.439
High SO	0.355	0.533	0.471	0.530
Not-high SO	0.687	0.634	0.585	0.405
High AA	0.628	0.801	0.280	0.268
Not-high AA	0.426	0.441	0.792	0.615
High PP	0.653	0.753	0.394	0.340
Not-high PP	0.428	0.485	0.715	0.607
High HE	0.560	0.679	0.431	0.392
Not-high HE	0.498	0.539	0.646	0.524
High GC	0.584	0.748	0.354	0.340
Not-high GC	0.485	0.500	0.738	0.571
High SI	0.607	0.790	0.263	0.256
Not-high SI	0.429	0.437	0.785	0.599
High SE	0.625	0.755	0.320	0.290
Not-high SE	0.412	0.447	0.730	0.593

### 4.2 Sufficiency analysis of conditional configurations

Since no single condition qualifies as a necessary condition, this paper further employs fuzzy-set qualitative comparative analysis (fsQCA) to examine combinations of multiple conditions in order to identify various configurational pathways for achieving a high level of national fitness public service governance. Following QCA research conventions, the case frequency threshold was set at 1, the consistency threshold at 0.85, and the PRI consistency threshold at 0.75. The analysis results are presented in Table 5.

**Table 5:** Results of configuration pathway analysis for high-level national fitness public service governance performance.

Dimension	Condition	H1	H2	H3	H4	H5	H6	H7	H8
Wuli	SC	•	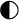	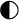	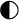	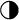	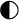	•	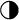
Wuli	FE	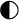	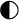	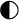	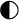	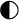	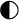	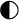	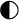
Wuli	SO	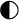	⊗	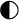	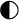	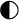	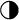	⊗	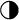
Shili	AA	•	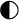	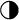	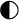	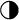	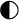	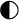	•
Shili	PP		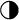	•	•	•	•	•	⊗
Shili	HE	•	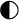	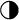	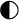	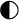	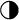	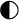	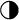
Renli	GC	•	•	⊗	⊗	⊗	⊗	•	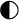
Renli	SI	•	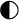	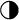	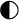	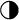	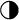	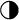	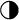
Renli	SE	•	•	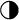	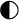	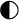	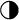	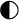	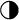
Raw coverage		0.271	0.049	0.071	0.148	0.061	0.069	0.060	0.065
Unique coverage		0.230	0.025	0.043	0.124	0.030	0.040	0.036	0.033
Consistency		0.939	0.945	0.962	0.963	0.973	0.872	0.938	0.928
Overall coverage		0.640							
Overall consistency		0.954							

• = core condition present, ⊗ = core condition absent; 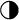 = peripheral condition present, 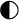 = peripheral condition absent; blank = condition may or may not be present.

To further examine causal asymmetry, this study conducted a configurational analysis of non-high-level national fitness public service governance effectiveness. As shown in Table 6, four pathways to non-high-level effectiveness (NH1–NH4) were identified, with an overall consistency of 0.916 and an overall coverage of 0.384. These findings indicate that non-high-level governance effectiveness arises from configurations of conditions that differ from those associated with high-level governance effectiveness; therefore, its formation mechanism is not simply the inverse of the high-level pathways.

**Table 6:** Results of configuration pathway analysis for non-high-level national fitness public service governance performance.

Dimension	Condition	NH1	NH2	NH3	NH4
Wuli	SC	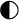	•	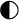	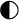
Wuli	FE	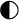	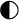	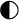	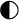
Wuli	SO	•	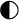	⊗	⊗
Shili	AA	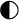	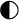	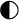	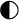
Shili	PP	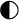	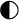	⊗	⊗
Shili	HE	⊗	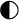	•	•
Renli	GC	⊗	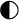	⊗	⊗
Renli	SI	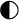	⊗	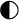	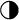
Renli	SE		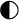	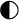	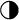
Raw coverage		0.217	0.084	0.067	0.078
Unique coverage		0.194	0.052	0.039	0.060
Consistency		0.867	0.949	0.967	0.972
Overall coverage		0.384			
Overall consistency		0.916			

• = core condition present, ⊗ = core condition absent; 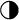 = peripheral condition present, 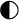 = peripheral condition absent; blank = condition may or may not be present.

From the overall results, a total of eight equivalent pathways for achieving high-level public service governance were identified. The overall solution consistency was 0.954 and the overall solution coverage was 0.640, indicating that the extracted conditional combinations have strong explanatory power for the outcome variable. The consistency of each individual pathway exceeded 0.872, with most pathways exceeding 0.9, suggesting high robustness in explaining high-level public service governance. Further examining the conditional configuration characteristics from the WSR perspective, although the configurational pathways differ in their specific combinations, they collectively exhibit a salient feature of multi-element synergistic driving: from the Wuli dimension, resource supply elements play a foundational supporting role in most pathways. The synergistic presence of variables such as venue facility level, fiscal guarantee intensity, and social organization scale reflects the foundational supporting role of physical resource adequacy and allocation structure in governance effectiveness. In some configurational pathways, venue facility level appears as a core condition, indicating that the completeness of infrastructure directly affects the accessibility and stability of national fitness public services, serving as a critical foundation for ensuring orderly service provision; from the Shili dimension, institutional supply and operational mechanisms occupy key positions in some pathways. Variables such as institutional completeness, policy supply intensity, and activity frequency occupy key positions in some pathways, reflecting the normative and enabling role of institutional logic on governance behavior. Shili elements, by building stable rule systems and operational mechanisms, reduce transaction costs within the system, enhance the predictability and coordination of multi-actor behavior, and thereby compensate, to a certain extent, for the constraints imposed by insufficient Wuli resource endowments; from the Renli dimension, governance actor capacity plays a critical transformative role in the pathways. The synergistic emergence of variables such as government service capacity, the number of social sports instructors, and the number of sports system employees reflects the pivotal role of government execution capacity, specialized human capital, and governance actor participation in the performance transformation process. Social sports instructors and sports system employees, as specialized human capital, help activate the utilization efficiency of Wuli resources, enhance the execution effectiveness of institutional operations, and facilitate the transformation of governance resources and institutional advantages into tangible national fitness public service governance outcomes.

What merits deeper exploration is that across all high governance effectiveness configurational pathways, “per capita mass sports expenditure” consistently appears as a peripheral absence. At first glance, this finding appears to contradict the conventional wisdom that “fiscal input is the foundational guarantee for public services,” but it in fact embodies a deeper governance logic. In conjunction with the necessity analysis, “not-high per capita mass sports expenditure” had a consistency of 0.940 and a coverage of 0.601; moreover, across the four pathways for non-high governance effectiveness, this variable likewise uniformly appeared as a peripheral absence, and “not-high per capita mass sports expenditure” as a necessary condition had a consistency of 0.916. This indicates that insufficient fiscal input is a sufficient condition for low governance effectiveness (a threshold effect), yet sufficient fiscal input is not a sufficient condition for high governance effectiveness (asymmetry).

This “asymmetric causality” characteristic can be elucidated at three levels:

First, the “institutional absorption threshold” of fiscal input. The impact of fiscal input on governance effectiveness is not linearly increasing but rather follows the law of diminishing marginal utility. Once per capita sports expenditure reaches a certain basic threshold, further increases in fiscal input, if lacking matching institutional absorption capacity (such as scientific project design, efficient implementation mechanisms, and precise needs identification), are difficult to convert into effective public service outputs. In the high governance effectiveness configurations, although per capita mass sports expenditure is not a core condition, the synergistic presence of the number of national fitness projects in the Wuli dimension and the number of social sports instructors and sports system employees in the Renli dimension forms a functional substitution of “physical capital + human capital” for monetary capital.

Second, the “compensatory mechanism” of substitutive elements. In high governance effectiveness configurations, when the level of fiscal expenditure is low, the effective allocation of other elements can compensate, to a certain degree, for the shortfall in funding. For instance, a high-density deployment of sports system employees can enhance the utilization efficiency of per-unit fiscal expenditure, and an adequate supply of social sports instructors can fill the funding gaps in facility maintenance and activity organization. This “functional substitution among elements” represents a typical complementarity logic in configuration analysis.

Third, the structural signal of governance phase transition. The retreat of fiscal input from a “core condition” to a “peripheral condition” carries a deeper implication: China's national fitness public service governance is undergoing a transition from “resource-driven” to “institution- and coordination-driven” models. In the early stages of governance system construction, the scale effect of fiscal input is significant; but when the governance system enters a relatively mature stage, structural factors such as the scientific quality of institutional design, the effectiveness of actor coordination, and the professionalism of human capital begin to play a more critical differentiating role. Consequently***, per capita mass sports expenditure*** generally appears as a peripheral absence in high-performance configurations. It should be noted that this does not imply that fiscal input is unimportant—indeed, in recent years the central government has continued to increase investment in national fitness through channels such as lottery public welfare funds—but rather indicates that under conditions where Shili and Renli elements are fully developed, the marginal utility of fiscal input diminishes, its foundational role can be partially substituted and compensated for, and further improvement in governance effectiveness depends more on the systemic optimization of element combinations than on the continuous escalation of any single resource.

In summary, the enhancement of national fitness public service governance effectiveness does not depend on a single-dimensional advantage but rather on the synergistic effects of multiple types of elements within the WSR system. The Wuli, Shili, and Renli elements in national fitness public service governance effectiveness exhibit pronounced multiple conjunctural causation characteristics, with different conditions forming diverse combinations in terms of resource elements, institutional supply, and governance capacity, jointly driving the realization and improvement of governance performance.

### 4.3 WSR-based configuration pathway pattern analysis

Building on the preceding configuration analysis, this paper integrates the “Wuli-Shili-Renli” (WSR) systems methodology to systematically synthesize and theoretically distill the multiple pathways for achieving high-level national fitness public service governance performance (Table 5). Although the configurational pathways differ in their specific conditional configurations, based on the dominant element structure and their operative mechanisms, the eight pathways (H1-H8) can be classified into three typical models: the Resource-Driven Model, the Institution-Driven Model, and the Collaborative Governance Model. These three models correspond respectively to the Wuli-dominant logic, the Shili-dominant logic, and the multi-dimensional synergistic logic within the WSR framework, reflecting the differentiated operative mechanisms of different elements in the governance process.

Type 1: Resource-Driven Model (H4).

This model is dominated by Wuli dimension elements, and its typical characteristic is the concentrated allocation of infrastructure and resource input elements. In pathway H4, the number of national fitness projects (SC) is present as a core condition, while the annual average number of special policies (PP) and the number of social sports instructors (SI) serve as peripheral conditions in a supporting role, presenting a structural feature of “resource foundation + institutional embeddedness.” Overall, this pathway type takes the adequate supply of resource elements as its premise and achieves effective improvement in governance performance by strengthening infrastructure and input guarantees.

Type 2: Institution-Driven Model (H2, H3, H5).

This model takes Shili dimension elements as its core, and its configurational characteristics are manifested in the dominant role of policy supply and institutional elements. In pathway H2, provincial government service capacity (GC) and the number of sports system employees (SE) serve as dual core conditions in a synergistic leading role, with the annual average number of special policies (PP) providing auxiliary support as a peripheral condition, while the number of national fitness projects (SC), per capita mass sports expenditure (FE), the number of sports venue institutions (AA), the number of public health education activities (HE), and the number of social sports instructors (SI) are all peripheral absences, and per capita sports social organizations (SO) is a core absence. The overall structure is characterized by “core capacity-core human resources-peripheral institutions” synergistic driving. In pathway H3, the annual average number of special policies (PP) plays the leading role as the sole core condition, with the number of sports venue institutions (AA), the number of public health education activities (HE), the number of social sports instructors (SI), and the number of sports system employees (SE) synergistically supporting as peripheral conditions, while the number of national fitness projects (SC), per capita mass sports expenditure (FE), and per capita sports social organizations (SO) are peripheral absences, and provincial government service capacity (GC) is a core absence. The overall structure is characterized by “institutional core dominance, multi-dimensional peripheral support, governance capacity absence.” In pathway H5, the annual average number of special policies (PP) plays the leading role as a core condition, with the number of sports venue institutions (AA) and the number of social sports instructors (SI) providing auxiliary support as peripheral conditions, while all remaining conditions appear as absences. The overall structure is characterized by “institutional core dominance, facility, and human resource peripheral synergy.” Overall, this pathway type achieves improvement in governance performance by compensating, to a certain extent, for differences in resource conditions through institutional supply and governance mechanism optimization.

Type 3: Collaborative Governance Model (H1, H6, H7, H8).

This model reflects the combined effects of Wuli, Shili, and Renli multi-dimensional elements, and its salient characteristic is the interactive embedding and systemic synergy of multiple types of conditions. Pathway H1 is the most representative case with the richest conditional configuration: six core conditions—the number of national fitness projects (SC), the number of sports venue institutions (AA), the number of public health education activities (HE), provincial government service capacity (GC), the number of social sports instructors (SI), and the number of sports system employees (SE)—are synergistically present, covering the three dimensions of Wuli facilities, Shili operations, and Renli capacity, forming a complete “resource supply-institutional support-actor capacity” synergistic system. In pathway H6, elements such as the number of national fitness projects, the number of public health education activities, the number of social sports instructors, and the number of sports system employees interact jointly, forming a structure of synergistic resource and human resource allocation; pathway H7 further superimposes provincial government service capacity and social sports instructors, exhibiting a multi-dimensional coupling characteristic of resources, capacity, and human resources; pathway H8 similarly forms a relatively complete synergistic system based on the joint presence of multiple types of elements. This model demonstrates that the improvement of national fitness public service governance performance depends on the coupling and linkage among multi-dimensional elements, representing a typical systemic governance pathway.

Taken together, the above three governance models reveal the intrinsic mechanisms of national fitness public service governance performance improvement from different dimensions: the Resource-Driven Model relies on the continuous investment and accumulation of Wuli elements, the Institution-Driven Model achieves pathway substitution through institutional supply and governance mechanism optimization, and the Collaborative Governance Model realizes systemic optimization through multi-dimensional element integration. This not only confirms the applicability of the WSR methodology in explaining complex public governance issues but also further demonstrates that national fitness public service governance possesses significant equifinality and systemic complexity. Different regions should select development pathways matched to their own resource endowments, institutional environments, and governance capacity foundations, thereby achieving sustained optimization and improvement in public service governance performance.

### 4.4 Robustness check

To ensure the reliability of the research findings, this paper conducted multiple robustness checks. Following established QCA research conventions, sensitivity analyses were performed across three dimensions: adjusting the consistency threshold, adjusting the PRI consistency threshold, and adjusting the case frequency threshold (19, 20).

(1) Adjusting the consistency threshold. The consistency threshold in the original analysis was raised from 0.80 to 0.85, and the truth table analysis and configuration extraction were re-conducted. The results showed that the number of configurations, core condition configurations, and coverage and consistency indicators did not undergo substantive changes, indicating that the initial configuration results are not sensitive to the setting of the consistency threshold.
(2) Adjusting the PRI consistency threshold. The PRI consistency threshold was raised from 0.75 to 0.80, and the configuration analysis was re-conducted. The results indicated that the configuration structure remained highly consistent with the original results; the core conditions of each pathway did not change, and the overall conclusions remained robust.
(3) Adjusting the case frequency threshold. The case frequency threshold was raised from 1 to 2, yielding new configuration results (Q1 and Q2), as shown in Table 7. Comparison with the original configurations revealed that Q1 has a subset relationship with the original configuration H2, and Q2 has a subset relationship with the original configuration H4; moreover, the core condition configurations of Q1 and Q2 remained consistent with their corresponding original configurations. This indicates that after raising the case frequency threshold, the core structure of the original configurations was preserved, further validating the robustness of the research findings.

**Table 7:** Results of configuration analysis after adjusting the case frequency threshold.

Dimension	Condition	Q1	Q2
Wuli	SC	•	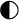
Wuli	FE	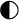	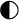
Wuli	SO	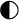	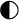
Shili	AA	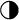	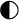
Shili	PP		•
Shili	HE	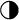	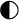
Renli	GC	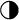	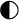
Renli	SI	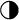	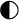
Renli	SE	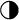	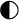
Raw coverage		0.271	0.148
Unique coverage		0.266	0.143
Consistency		0.939	0.963
Overall coverage		0.414	
Overall consistency		0.951	

• = core condition present, ⊗ = core condition absent; 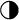 = peripheral condition present, 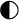 = peripheral condition absent; blank = condition may or may not be present.

This test demonstrates that the core conditional combination patterns driving high governance effectiveness are stable under different analytical standards, and the critical conditions constituting the “core recipe” for high effectiveness and their combinatorial logic did not undergo essential change. This strongly supports the robustness of this study's main findings, namely that the identified multiple configurational pathways are not artifacts incidentally generated by specific parameter settings but rather reflect genuine, reliable causal patterns in government national fitness public service governance.

### 4.5 Regional heterogeneity

Given the significant differences in regional development levels and public service supply capacities across China, this paper further conducts configuration analyses for the eastern, central, and western regions respectively from a regional perspective, in order to examine the differential pathways for achieving national fitness public service governance performance under different regional contexts. The analysis results are presented in Table 8 below.

**Table 8:** Results of high-level configuration analysis by region.

Dimension	Condition	Eastern					Central				Western			
		E1	E2	E3	E4	E5	M1	M2	M3	M4	W1	W2	W3	W4
Wuli	SC	•	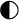	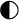	•	•	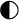	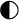	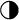	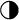	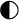	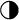	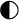	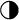
Wuli	FE	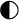	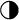	⊗	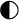	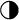	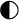	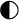	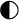	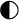	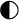	•	•	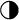
Wuli	SO	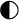	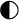	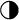	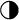	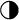	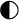	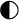	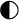	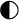	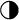	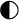	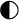	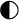
Shili	AA	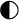	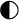	•	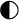	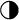	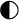	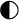	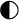	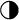	•	•	•	•
Shili	PP	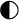	⊗	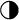	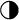	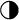	•	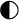	•	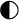	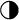	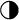	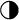	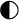
Shili	HE	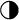	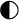	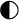	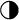	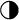	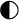	•	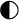	•	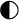	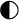	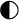	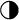
Renli	GC	⊗	•	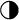	⊗	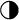	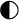	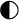	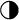	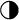	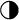	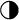	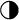	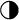
Renli	SI	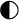	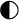	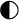	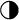	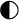	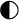	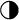	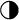	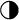	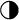	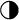	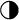	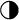
Renli	SE	⊗	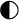	⊗	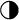	⊗	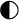	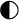	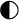	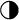	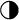	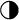	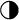	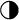
Raw coverage		0.159	0.202	0.167	0.213	0.155	0.150	0.093	0.170	0.345	0.150	0.194	0.130	0.201
Unique coverage		0.071	0.117	0.077	0.124	0.068	0.141	0.084	0.161	0.343	0.127	0.125	0.092	0.157
Consistency		0.843	0.866	0.889	0.910	0.881	0.894	0.825	0.941	0.965	0.966	1.000	0.987	0.991
Overall coverage		0.563					0.739 0.569							
Overall consistency		0.934					0.954 0.988							

• = core condition present, ⊗ = core condition present; 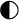 = peripheral condition absent, 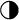 = peripheral condition present; blank = condition may or may not be present.

As can be seen from Table 8, the eastern, central, and western regions exhibit significant differences in the number of pathways, element allocation patterns, and dominant driving mechanisms, displaying a clear gradient differentiation pattern across regions.

The eastern region has the richest variety of pathway types (E1-E5), dominated by collaborative governance pathways. Their common structural feature is that core conditions from all three categories—Wuli, Shili, and Renli—are often simultaneously present, with the largest number of conditional elements included in the pathways and the highest degree of coupling. Pathway E1 has the number of national fitness projects (SC) as a core condition and the number of public health education activities (HE) as peripheral support, presenting a hierarchical structure of “core resources-peripheral activities”; pathway E4 has SC as the core, the number of social sports instructors (SI) as peripheral assistance, and HE simultaneously supplementing as a peripheral condition, presenting a structural feature of “resource dominance, human resource, and activity synergy.” Pathways E2, E3, and E5 further superimpose provincial government service capacity (GC) and related policy supply (PP), presenting a composite structure of institutional supply and governance capacity embeddedness. Among these, in pathways E2 and E5, per capita mass sports expenditure (FE) also exists as a peripheral condition; although it has not risen to core status, this indicates that in some pathways in the eastern region, fiscal input still constitutes a foundational auxiliary constraint, forming multi-dimensional linkages with institutional and capacity elements. Overall, the eastern region has already transitioned from single-element driving to multi-dimensional deep synergistic driving, with the highest pathway complexity.

The central region's pathway structure is relatively balanced (M1–M4), characterized by the coexistence of resource-driven and institution-driven dual tracks, transitioning toward collaborative governance. Pathway M1 has the annual average number of special policies (PP) as the sole core condition, while pathway M2 has the number of public health education activities (HE) as the sole core condition, both presenting a typical single-dimensional driving pattern; pathways M3 and M4, however, simultaneously include conditions such as the number of sports venue institutions, the number of social sports instructors, and the number of sports system employees, indicating that Renli elements are gradually embedding into the governance structure. The central region's pathways encompass both resource support and a progressive strengthening of institutional and human resource elements in their element allocation, reflecting an intermediate transitional state moving from resource-driven toward collaborative governance.

The western region has the most concentrated pathway types (W1–W4), characterized by single-dimensional element driving and simplified pathway structures. The number of core conditions in each pathway is relatively small, and most are dominated by a single Wuli element (number of national fitness projects, per capita mass sports expenditure) or a single Shili element (number of sports venue institutions, annual average number of special policies), with the degree of Renli element participation being significantly lower than in the eastern and central regions. In pathways W2 and W3, per capita mass sports expenditure appears as a core condition while elements from other dimensions are absent, reflecting that the western region is still in the resource accumulation stage; although pathways W1 and W4 introduce relevant policy and social sports instructor elements, the overall degree of synergy is limited, representing a “limited synergy” form.

It is noteworthy that “per capita mass sports expenditure” (FE), which is generally a core absence in the national configuration (Table 5), instead appears as a core condition in western pathways W2 and W3. This contrast precisely confirms the core proposition of regional heterogeneity: the functional status of the same element undergoes structural changes at different developmental stages. In the western region's resource accumulation stage, fiscal input has not yet crossed the basic threshold and remains a bottleneck constraint on governance effectiveness improvement; whereas in the eastern and central regions, the marginal contribution of fiscal input has already been overshadowed by the functional substitution of institutional and Renli elements, causing it to retreat to a peripheral condition. This finding further indicates that the effectiveness of governance pathways is highly context-dependent, and the configurational patterns identified at the national level cannot be simply extrapolated to specific regions.

The evolutionary logic of inter-regional structural differences: from east to west, pathway complexity decreases progressively (eastern multi-dimensional coupling > central dual-track coexistence > western single-dimensional driving), the degree of Renli element participation declines progressively, and the dominant position of Wuli elements rises progressively. This gradient pattern reflects that China's national fitness public service governance is undergoing a phased evolution from “resource foundation- institutional improvement- systemic synergy,” with different regions situated at different evolutionary stages due to structural differences in resource endowments, institutional environments, and governance capacities. Meanwhile, a nested relationship of “common patterns-contextual differences” emerges between regional configurations and the national configuration: the three models identified at the national level—Resource-Driven, Institution-Driven, and Collaborative Governance—are all reflected in each region, yet the regional configurations exhibit significant localized adaptation characteristics in the specific forms of element allocation.

### 4.6 Discussion

The core finding of this study—that national fitness public service governance effectiveness is achieved through three equivalent pathways, namely the “Resource-Driven Model,” the “Institution-Driven Model,” and the “Collaborative Governance Model,” and that pathway selection exhibits significant regional context-dependence—engages in dialogue with existing theories at multiple levels and contributes new theoretical insights.

#### 4.6.1 Dialogue with public service governance theory

Traditional public service governance research has long been characterized by a “structure-agency” debate: one side emphasizes the decisive role of institutional structures and resource supply (e.g., the New Public Management theory's focus on “input-output” efficiency), while the other highlights the central position of actor agency and governance capacity (e.g., governance theory's emphasis on “interaction-coordination”). The findings of this study provide empirical evidence for transcending this dichotomy: none of the three categories of elements constitutes a necessary condition for governance effectiveness, yet all three can produce high effectiveness through specific combinations. This implies that no a priori hierarchical relationship exists among Wuli, Shili, and Renli; their relative importance depends on the specific governance context. This finding supports the proposition of “contextualized effectiveness” in public governance theory—governance success depends not on some universal best practice but on the degree of fit between element allocation and contextual demands.

#### 4.6.2 Revision and extension of the “Resource-Based View”

The finding of “core absence of fiscal input” in this study constitutes an important revision to the “resource-based view” in public service research. The resource-based view emphasizes that resource endowments are the fundamental determinants of organizational performance, but this study demonstrates that once the governance system reaches a basic level of maturity, the marginal contribution of resources to performance weakens, and the complementary configuration of institutional and capacity elements becomes the primary source of performance differentiation. This resonates with the concept of “institutional congestion” in institutional economics—when institutional absorption capacity is insufficient, newly added resources may generate efficiency losses rather than performance gains due to excessively long “input-transformation” chains. From this, a mid-range theoretical proposition can be distilled: there exists a “Wuli element threshold effect” in public service governance effectiveness—Wuli resources constitute a “necessary condition set” rather than a “sufficient condition set” for governance performance; their core role lies in setting the lower bound of performance, while the synergistic allocation of Shili and Renli elements determines the upper bound. It is worth emphasizing that this finding does not imply that fiscal input is “excessive in aggregate” or “no longer important”; rather, it reveals that the operative mechanism of fiscal input exhibits phase-specific characteristics: in the early stages of governance system construction, the scale of input determines the breadth of service coverage; when the governance system enters a relatively mature stage, the marginal utility of input diminishes, and its effectiveness transformation depends more on the supporting levels of institutional absorption capacity and human capital execution capacity. Therefore, the policy implication is not “reduce input” but “optimize allocation”—while maintaining necessary input, greater attention should be paid to the synergistic matching of input with institutions and human resources.

#### 4.6.3 Connection with configuration research literature

The three pathway models identified in this study are consistent with the findings of Xue et al. (11) and other scholars, all confirming that policy instruments or governance elements exhibit configurational effects in public services. However, this paper further advances existing research in two respects: first, it extends the analytical framework from a single policy instrument dimension to the three-dimensional “Wuli-Shili-Renli” system, revealing the cross-dimensional substitution and complementarity mechanisms among elements; second, through regional heterogeneity analysis, it reveals the context-dependence of equivalent pathways—pathways likewise classified as “Resource-Driven Model” exhibit significant differences in specific element allocation between eastern and western regions, indicating that configurational pathways possess not only “cross-case equifinality” but also “cross-context differentiation,” the latter having received less attention in existing QCA research.

#### 4.6.4 Theoretical response to real-world challenges

While identifying multiple pathways to success, it is necessary to confront the real-world challenges facing current national fitness public service governance, in order to more comprehensively assess the policy implications of this study's findings. First, cross-departmental coordination mechanisms remain inadequate; departments of sports, health, education, and civil affairs have yet to form effective synergy in policy objectives, resource allocation, and evaluation systems. This partially explains why the “Institution-Driven Model” in the central and western regions is still dominated by a single institutional element rather than multi-institutional synergy. Second, service provision quality and facility utilization efficiency need improvement; in some regions, public sports venues suffer from the phenomenon of “built but unused” and “disconnect between management and use,” which precisely corroborates this study's core finding—Wuli resources themselves do not automatically generate governance effectiveness; their utility depends on the supporting levels of Shili and Renli elements. Third, there remains a significant gap in urban-rural facility coverage rates; a certain gap in urban-rural facility coverage still exists in some regions, meaning that the “Resource-Driven Model” still holds phased applicability value in western rural areas, but in the long term a transition toward the “Collaborative Governance Model” is necessary. The existence of these challenges does not undermine the study's findings; on the contrary, they confirm from the reverse perspective the necessity of multi-element synergistic allocation—it is precisely because the limitations of single-element driving have repeatedly manifested in practice that the WSR three-dimensional synergistic governance logic carries urgent practical significance.

## 5 Conclusions and implications

### 5.1 Research conclusions

This study, based on the WSR systems methodology, constructs an integrated analytical framework and employs fuzzy-set qualitative comparative analysis (fsQCA) to conduct a configurational path analysis of the public sports service governance effectiveness of 31 provincial-level governments in China. Through a systematic examination of the complex interactive relationships among multiple antecedent conditions, this study draws the following core conclusions:

First, the formation of high governance effectiveness does not rely on any single necessary condition, displaying significant multiple conjunctural causation. The necessity analysis results indicate that neither financial investment, infrastructure provision, social organization foundation, nor policy supply, government capacity, and professional human resource allocation individually constitutes a necessary condition for high-level governance effectiveness of National Fitness public services. This suggests that governance effectiveness is not driven linearly by any single “key variable” but rather results from the combined effect of multiple types of elements through differentiated combinations in different contexts, embodying typical equifinality. This finding empirically refutes “single-factor determinism” and “input-scale determinism,” revealing the complexity and nonlinear characteristics of public service governance systems.

Second, the synergistic configuration of the three WSR dimensions constitutes the fundamental mechanism for enhancing the governance effectiveness of National Fitness public services. The configurational analysis results show that Wuli elements provide foundational support for the delivery of National Fitness public services, Shili elements play a guiding role through institutional supply and operational mechanism optimization, and Renli elements play a critical role in governance execution and performance transformation. Different configurations achieve enhanced governance effectiveness through differentiated combinations of the three types of elements, exhibiting significant element complementarity—that is, when elements of one dimension are relatively insufficient, elements of other dimensions can, to a certain extent, play a substitutive role. However, if elements of any dimension are absent over the long term, it is difficult to form a stable and efficient governance structure. This result empirically validates the applicability of WSR theory in public service governance, indicating that the governance effectiveness of National Fitness public services is essentially a systemic output of the synergistic interaction of multi-dimensional elements.

Third, the three types of path structures—“Resource-Driven Model,” “Institution-Driven Model,” and “Collaborative Governance Model”—reflect differentiated element-dominance logic. First, the “Resource-Driven Model” is dominated by Wuli-dimension elements, emphasizing the foundational role of infrastructure provision in enhancing governance effectiveness. In this type of path, Wuli elements such as National Fitness Projects (SC) occupy a key position, providing solid material support for governance effectiveness enhancement by strengthening public service delivery capacity, reflecting typical factor-input-driven characteristics. Second, the “Institution-Driven Model” is centered on Shili-dimension elements, highlighting the guiding and regulatory role of policy supply and institutional arrangements in the governance process. In this type of path, Annual Average Special Policies (PP) plays a core dominant role, while elements such as Sports Venue Institutions (AA) and Public Health Education Activities (HE) provide auxiliary support as peripheral conditions. By optimizing the institutional environment and operational mechanisms, this model compensates for resource endowment disparities to a certain extent and achieves effective enhancement of governance effectiveness, reflecting institution-supply-driven characteristics. Third, the “Collaborative Governance Model” embodies the systemic coupling of Wuli, Shili, and Renli multi-dimensional elements and results from the synergistic interaction of multiple actors and multiple elements. Path H1 is particularly typical: six core conditions—National Fitness Projects (SC), Sports Venue Institutions (AA), Public Health Education Activities (HE), Provincial Government Service Capacity (GC), Social Sports Instructors (SI), and Sports System Employees (SE)—are simultaneously present, forming a complete “resource provision–institutional support–actor capacity” collaborative system. In addition, paths H6, H7, and H8 similarly exhibit characteristics of multi-dimensional element linkage, reflecting the evolutionary direction from single-factor-driven to systemic synergy-driven approaches. Overall, the three models respectively reveal the multiple realization mechanisms for enhancing the governance effectiveness of National Fitness public services from three dimensions: resource foundation, institutional supply, and systemic synergy.

Fourth, the realization of governance effectiveness in National Fitness public services exhibits significant regional heterogeneity and demonstrates phased evolutionary characteristics. The regional configurational analysis results indicate that the eastern region is dominated by the Collaborative Governance Model path, characterized by a high degree of multi-dimensional element coupling; the central region exhibits coexistence of resource-driven and institution-driven approaches, transitioning toward collaborative governance; and the western region is primarily dominated by resource-driven or institution-driven approaches, with relatively simplified path structures. At the same time, regional configurations reflect the differentiated realization modes of the three dominant models in different contexts. Overall, China's National Fitness public service governance path exhibits an evolutionary trend from resource-driven to institution-driven and then to collaborative governance, reflecting the structural differences among different regions in terms of development stage, resource endowment, and governance capacity.

### 5.2 Policy implications

Based on the above four core conclusions, this study proposes the following policy implications for enhancing the governance effectiveness of National Fitness public services:

(1) In response to the “no single necessary condition” finding: Establishing a factor combination assessment system and moving beyond single-indicator evaluation

This study finds that no single element constitutes a necessary condition for high governance effectiveness. This implies that performance assessment systems centered on single indicators such as “per capita venue area” and “fiscal investment growth rate” cannot accurately reflect the full picture of governance effectiveness. It is recommended that governments at all levels, in the evaluation mechanisms of the “15th Five-Year Plan” for National Fitness implementation—which has already commenced preparation—introduce a factor combination assessment framework that integrates three categories of indicators—Wuli (facilities, funding, organizations), Shili (institutions, coordination, operations), and Renli (government capacity, professional human resources, social participation)—into a comprehensive evaluation system, thereby avoiding crude evaluation approaches that judge success solely by “per capita venue area.” The focus of evaluation should shift from “whether inputs exist” to “whether the factor combination is rational and whether the allocation structure is optimized.”

(2) In response to the “WSR synergistic configuration” finding: Building a linkage mechanism of “Wuli as foundation—Shili as guidance—Renli as transformation”

This study confirms that the synergistic configuration of the three types of elements is the fundamental mechanism for enhancing governance effectiveness. Accordingly, the following recommendations are proposed: (1) In the Wuli dimension, infrastructure investment should be continuously increased, but the focus should shift from “incremental expansion” to “stock optimization”—improving the operational efficiency and service accessibility of existing facilities, and especially addressing facility gaps in underserved areas such as rural regions and old urban districts. (2) In the Shili dimension, efforts should be made to break down institutional barriers among sectors such as sports, health, education, and civil affairs, establishing cross-departmental policy coordination and resource integration mechanisms to enhance the systemic coherence and consistency of institutional supply. (3) In the Renli dimension, emphasis should be placed on strengthening the development of social sports instructors and grassroots sports management talent teams, improving training, certification, and incentive mechanisms, while simultaneously enhancing local government execution effectiveness through performance assessment and capacity training. The three types of elements should form linkages through institutional design—for example, at the planning stage of new facility construction, social sports instructors and operational management teams should be synchronously allocated to avoid problems such as “building without managing” or “management detached from utilization.”

(3) In response to the “three types of paths” and “regional heterogeneity” findings: Implementing differentiated and targeted policies for eastern, central, and western regions

The three models identified in this paper—“Resource-Driven Model,” “Institution-Driven Model,” and “Collaborative Governance Model”—exhibit significant context dependency:

The eastern region has largely entered the “Collaborative Governance Model” stage and should, on its existing foundation, focus on advancing institutional innovation and digital empowerment, exploring new governance models such as smart sports, precision services, and deep participation of social forces, thereby providing pioneering experience for the nation. The policy focus should shift from “incremental construction” to “stock optimization and quality enhancement.”

The central region is currently in the transition stage from “resource-driven” to “institution-driven” and should accelerate institutional supply and governance capacity building. While continuing to improve infrastructure, emphasis should be placed on strengthening policy innovation, cross-departmental coordination mechanisms, and professional talent allocation, thereby promoting the evolution of governance paths toward collaborative governance.

The western region remains predominantly driven by the “Resource-Driven Model” path and should prioritize consolidating infrastructure and fiscal guarantees, addressing resource gaps through central transfer payments, paired assistance, and other means. At the same time, conscious efforts should be made to cultivate social sports organizations and professional talent teams, creating conditions for a future leap to higher-level paths and avoiding the structural imbalance of “resources supplemented but institutions not keeping pace.”

(4) In response to the path diversity and dynamic evolution characteristics: establishing a data-driven dynamic monitoring and path optimization mechanism

Under a governance landscape characterized by multi-path coexistence and context dependency, localities should establish a normalized data-driven assessment system. Specifically: (1) Leveraging the National Fitness Information Service Platform, real-time monitoring should be conducted on indicators such as facility utilization efficiency, institutional implementation effectiveness, and public participation levels, forming a full-chain data system covering “process–output–outcome.” (2) A “monitoring–assessment–feedback–optimization” closed-loop mechanism should be established, embedding assessment results into the fiscal budgeting and policy adjustment process, and promptly and precisely addressing factor deficiencies and structural imbalances identified in the assessment. (3) A dynamic early warning mechanism for governance paths should be established: when governance effectiveness at a given stage approaches a bottleneck, resources should be directed toward domains and paths with significant effectiveness spillover, promoting the stepwise progression of governance paths from “resource-driven → institution-driven → collaborative governance,” thereby achieving long-term enhancement of governance effectiveness.

## Data Availability

The original contributions presented in the study are included in the article/Supplementary material, further inquiries can be directed to the corresponding author.
