# Supplementary material for: A configurational pathway study on enhancing the governance level of national fitness public services
Source: Front Public Health. 2026 Sep 10;14:1860547. doi: 10.3389/fpubh.2026.1860547 (PMC13603028; doi:10.3389/fpubh.2026.1860547)
Supplement: Supplementary file 1 [file Data_Sheet_1.pdf]

Appendix A-1 Raw Data and Calibrated Membership Scores by Province

Province	GE	SC	FE	SO	AA	PP	HE	GC	SI	SE
**Beijing**	0.778 (0.92)	1600 (0.83)	48.5 (0.63)	0.58 (0.51)	21.8 (0.93)	3.2 (0.87)	2980 (0.95)	87.5 (0.72)	228000 (0.95)	5900 (0.94)
**Tianjin**	0.770 (0.83)	1450 (0.65)	44.2 (0.48)	0.55 (0.41)	19.5 (0.78)	2.8 (0.72)	2650 (0.78)	85.2 (0.63)	195000 (0.73)	5200 (0.72)
**Hebei**	0.740 (0.48)	850 (0.22)	30.5 (0.28)	0.40 (0.18)	10.2 (0.32)	1.2 (0.15)	1400 (0.32)	81.5 (0.35)	120000 (0.28)	3800 (0.35)
**Shanxi**	0.728 (0.35)	780 (0.19)	28.0 (0.22)	0.38 (0.16)	8.5 (0.22)	0.8 (0.08)	1150 (0.22)	80.2 (0.28)	105000 (0.22)	3500 (0.28)
**Inner Mongolia**	0.720 (0.28)	500 (0.12)	26.5 (0.18)	0.36 (0.14)	7.2 (0.15)	0.5 (0.05)	1050 (0.18)	79.8 (0.22)	95000 (0.18)	3400 (0.25)
**Liaoning**	0.760 (0.72)	1200 (0.50)	40.0 (0.38)	0.52 (0.38)	16.5 (0.52)	2.2 (0.45)	2300 (0.52)	84.0 (0.52)	170000 (0.48)	4800 (0.52)
**Jilin**	0.725 (0.32)	750 (0.18)	27.5 (0.20)	0.37 (0.15)	8.0 (0.20)	0.6 (0.06)	1080 (0.20)	80.0 (0.25)	100000 (0.20)	3450 (0.26)
**Heilongjiang**	0.718 (0.25)	700 (0.16)	26.0 (0.17)	0.36 (0.14)	7.5 (0.17)	0.4 (0.04)	1020 (0.16)	79.7 (0.20)	93000 (0.17)	3350 (0.24)
**Shanghai**	0.782 (0.99)	1650 (0.99)	50.5 (0.71)	0.60 (0.57)	22.0 (0.96)	3.5 (0.94)	3100 (0.97)	88.5 (0.81)	232000 (0.97)	6000 (0.96)
**Jiangsu**	0.776 (0.95)	1550 (0.88)	47.0 (0.58)	0.57 (0.48)	21.0 (0.88)	3.0 (0.82)	2900 (0.88)	86.8 (0.68)	220000 (0.88)	5800 (0.88)
**Zhejiang**	0.779 (0.97)	1580 (0.92)	48.0 (0.60)	0.59 (0.54)	21.5 (0.92)	3.3 (0.90)	2950 (0.92)	87.0 (0.70)	225000 (0.92)	5850 (0.92)

Province	GE	SC	FE	SO	AA	PP	HE	GC	SI	SE
**Anhui**	0.745 (0.52)	950 (0.26)	34.0 (0.32)	0.44 (0.24)	12.0 (0.38)	1.6 (0.22)	1600 (0.38)	82.0 (0.38)	130000 (0.32)	4000 (0.40)
**Fujian**	0.750 (0.58)	1050 (0.32)	36.5 (0.35)	0.47 (0.28)	14.0 (0.45)	1.8 (0.28)	1800 (0.45)	82.5 (0.42)	140000 (0.36)	4200 (0.44)
**Jiangxi**	0.735 (0.42)	820 (0.20)	29.5 (0.25)	0.39 (0.17)	9.5 (0.28)	1.0 (0.10)	1300 (0.28)	81.0 (0.32)	115000 (0.25)	3700 (0.32)
**Shandong**	0.765 (0.78)	1300 (0.58)	42.0 (0.42)	0.53 (0.40)	17.5 (0.60)	2.5 (0.55)	2400 (0.58)	84.5 (0.55)	180000 (0.52)	5000 (0.58)
**Henan**	0.742 (0.50)	880 (0.23)	31.5 (0.30)	0.41 (0.20)	11.0 (0.34)	1.4 (0.18)	1500 (0.34)	81.8 (0.36)	125000 (0.30)	3900 (0.38)
**Hubei**	0.748 (0.55)	1000 (0.28)	35.5 (0.34)	0.46 (0.26)	13.5 (0.42)	1.7 (0.25)	1700 (0.42)	82.2 (0.40)	135000 (0.34)	4100 (0.42)
**Hunan**	0.743 (0.50)	920 (0.25)	33.0 (0.30)	0.43 (0.22)	11.5 (0.36)	1.5 (0.20)	1550 (0.36)	81.9 (0.37)	128000 (0.31)	3950 (0.39)
**Guangdong**	0.780 (0.98)	1620 (0.96)	49.5 (0.67)	0.59 (0.54)	21.8 (0.93)	3.4 (0.92)	3050 (0.96)	88.0 (0.76)	230000 (0.96)	5950 (0.95)
**Guangxi**	0.716 (0.24)	680 (0.15)	25.5 (0.16)	0.35 (0.13)	7.0 (0.14)	0.3 (0.03)	1000 (0.15)	79.6 (0.20)	90000 (0.16)	3320 (0.23)
**Hainan**	0.712 (0.20)	610 (0.13)	24.5 (0.14)	0.34 (0.12)	6.8 (0.13)	0.2 (0.02)	980 (0.14)	79.5 (0.18)	88000 (0.15)	3300 (0.22)
**Chongqing**	0.741 (0.48)	900 (0.24)	32.0 (0.28)	0.42 (0.21)	10.8 (0.33)	1.3 (0.16)	1450 (0.32)	81.6 (0.35)	122000 (0.28)	3850 (0.37)
**Sichuan**	0.738	860 (0.22)	30.0	0.40	10.0	1.1	1350	81.2	118000	3750

Province	GE	SC	FE	SO	AA	PP	HE	GC	SI	SE
	(0.45)		(0.26)	(0.18)	(0.30)	(0.12)	(0.30)	(0.33)	(0.26)	(0.34)
**Guizhou**	0.710 (0.18)	550 (0.12)	24.0 (0.13)	0.33 (0.11)	6.5 (0.12)	0.1 (0.01)	950 (0.13)	79.4 (0.17)	85000 (0.14)	3280 (0.21)
**Yunnan**	0.713 (0.22)	600 (0.13)	24.8 (0.15)	0.34 (0.12)	6.9 (0.14)	0.2 (0.02)	990 (0.14)	79.5 (0.18)	89000 (0.15)	3310 (0.22)
**Tibet**	0.705 (0.12)	400 (0.08)	22.0 (0.10)	0.31 (0.09)	6.0 (0.10)	0.0 (0.01)	900 (0.10)	79.2 (0.15)	80000 (0.12)	3250 (0.20)
**Shaanxi**	0.722 (0.30)	720 (0.17)	27.0 (0.19)	0.36 (0.14)	7.8 (0.18)	0.5 (0.05)	1060 (0.18)	79.9 (0.23)	98000 (0.19)	3420 (0.25)
**Gansu**	0.707 (0.15)	480 (0.10)	23.0 (0.11)	0.32 (0.10)	6.3 (0.11)	0.0 (0.01)	920 (0.11)	79.3 (0.16)	82000 (0.13)	3260 (0.20)
**Qinghai**	0.706 (0.14)	430 (0.09)	22.5 (0.10)	0.31 (0.09)	6.1 (0.10)	0.0 (0.01)	910 (0.11)	79.2 (0.15)	81000 (0.12)	3255 (0.20)
**Ningxia**	0.708 (0.16)	460 (0.10)	23.5 (0.12)	0.32 (0.10)	6.4 (0.12)	0.1 (0.01)	940 (0.12)	79.3 (0.16)	83000 (0.13)	3270 (0.21)
**Xinjiang**	0.711 (0.19)	520 (0.11)	25.0 (0.15)	0.33 (0.11)	6.6 (0.13)	0.1 (0.01)	960 (0.13)	79.4 (0.17)	86000 (0.14)	3290 (0.22)

Note: The calibration function is the membership score. Calibration anchors are shown in Table 3-3.$=1/\left(1+e^{-k\cdot(X-\text{交叉点})}\right)$ $k=\ln(99)/(\text{完全隶属}-\text{交叉点})$

Appendix A-2 Descriptive Statistics of the Three Sub-Indicators of GE

Sub-Indicators	Mean	SD	Min	Max
Per Capita Sports Venue Area (m^2 per capita)	2.58	0.45	1.85	4.32
Proportion of Townships Covered by Public Sports Venues (%)	62.3	18.7	28.5	98.0
Resident Satisfaction Score	78.5	5.2	68.0	89.5
National Physical Fitness Compliance Rate (%)	88.2	3.5	81.0	94.5
Proportion of Population Regularly Participating in Physical Exercise (%)	37.8	6.9	28.5	51.2

Appendix B Truth Table

Configuration No.	SC	FE	SO	AA	PP	HE	GC	SI	SE	No. of Cases	Raw Consistency	PRI Consistency	Outcome
1	1	0	0	1	—	1	1	1	1	5	0.939	0.921	1
2	0	0	0	0	1	0	1	0	1	2	0.945	0.918	1
3	0	0	0	1	1	1	0	1	1	2	0.962	0.935	1
4	0	0	0	0	1	0	0	0	0	3	0.963	0.942	1
5	0	0	0	1	1	0	0	1	0	2	0.973	0.956	1
6	1	0	1	0	1	1	0	1	1	2	0.872	0.835	1
7	1	0	0	0	1	0	1	1	0	2	0.938	0.912	1
8	1	0	1	1	0	1	0	1	1	2	0.928	0.905	1
9	0	0	0	0	1	0	0	0	1	1	0.894	0.862	1
10	0	0	0	0	0	1	0	0	0	1	0.825	0.788	0
11	1	0	0	0	0	0	0	0	0	1	0.867	0.832	0
12	0	0	1	0	0	0	0	0	—	1	0.949	0.925	0
13	0	0	0	0	0	1	0	0	0	1	0.967	0.948	0
14	0	0	0	0	0	1	0	1	1	1	0.972	0.955	0
15	0	0	0	0	0	1	0	0	0	1	0.966	0.944	0

Configuration No.	SC	FE	SO	AA	PP	HE	GC	SI	SE	No. of Cases	Raw Consistency	PRI Consistency	Outcome
16	0	0	0	1	1	1	0	0	1	1	0.958	0.932	1
17	0	0	0	0	1	0	0	1	1	1	0.944	0.915	1
18	0	0	1	0	1	1	0	1	0	1	0.937	0.908	1
19	0	0	0	1	0	0	1	0	1	1	0.921	0.887	1
20	1	0	0	0	0	1	0	1	0	1	0.896	0.858	1
21	0	0	1	1	0	0	0	1	0	1	0.883	0.842	1
22	0	0	0	0	0	1	1	0	0	1	0.978	0.960	0
23	0	0	1	0	0	0	1	0	0	1	0.971	0.952	0
24	0	0	0	1	0	0	0	1	0	1	0.965	0.943	0
25	0	1	0	0	0	0	0	0	1	1	0.959	0.935	0
26	1	0	0	0	0	0	1	0	0	1	0.952	0.927	0
27	0	0	0	0	1	0	1	0	0	1	0.946	0.919	0
28	0	0	0	0	0	0	0	1	1	1	0.933	0.902	0
29	0	1	0	0	0	0	0	1	0	1	0.925	0.891	0
30	0	0	0	0	0	0	0	0	1	1	0.918	0.882	0

Note: Configuration Nos. 1–8 correspond to Paths H1–H8 in Table 4-2 (high governance effectiveness); No. 9 represents other high-consistency configurations supported by cases but not

meeting the core condition configuration criteria; Nos. 10–15 are representative configurations with low governance effectiveness (see Table 4-3 for the full set of non-high configurations); the six "1"-outcome configurations among Nos. 16–21 complement the core paths H1–H8 in Table 4-2, together forming the complete set of high governance effectiveness configurations (8 core paths + 6 peripheral paths); the nine "0"-outcome configurations among Nos. 22–30, together with Paths NH1–NH4 in Table 4-3, explain the formation mechanism of non-high governance effectiveness. In the configurations, "1" indicates that the condition is present (membership > 0.5), "0" indicates that the condition is absent (membership < 0.5), and "—" indicates that the condition is eliminated in the parsimonious solution (i.e., "may or may not be present"). The number of cases refers to the number of provinces satisfying the configuration condition combination. An outcome of "1" indicates that the configuration leads to high governance effectiveness, and "0" indicates non-high governance effectiveness.

Appendix C-1 Provincial Cases and Membership Scores Corresponding to Each Configuration Path

Province	H1 Membership	H2 Membership	H3 Membership	H4 Membership	H5 Membership	H6 Membership	H7 Membership	H8 Membership	Primary Path
Beijing	0.92	0.38	0.45	0.42	0.35	0.48	0.52	0.40	H1
Tianjin	0.35	0.42	0.38	0.30	0.28	0.32	0.36	0.30	—
Hebei	0.22	0.28	0.25	0.62	0.20	0.30	0.35	0.28	H4
Shanxi	0.18	0.20	0.22	0.25	0.18	0.22	0.20	0.20	—
Inner Mongolia	0.15	0.18	0.16	0.20	0.14	0.16	0.18	0.15	—
Liaoning	0.42	0.45	0.40	0.48	0.35	0.42	0.44	0.38	—
Jilin	0.18	0.22	0.20	0.22	0.16	0.20	0.22	0.18	—
Heilongjiang	0.15	0.18	0.16	0.18	0.14	0.16	0.18	0.15	—
Shanghai	0.99	0.42	0.48	0.38	0.32	0.45	0.40	0.35	H1
Jiangsu	0.88	0.52	0.46	0.72	0.38	0.50	0.55	0.48	H1
Zhejiang	0.92	0.45	0.68	0.65	0.42	0.55	0.48	0.42	H1
Anhui	0.32	0.30	0.42	0.38	0.62	0.40	0.35	0.38	H5
Fujian	0.28	0.32	0.58	0.35	0.38	0.56	0.30	0.32	H3/H6
Jiangxi	0.20	0.22	0.25	0.28	0.22	0.24	0.22	0.24	—

Province	H1 Membership	H2 Membership	H3 Membership	H4 Membership	H5 Membership	H6 Membership	H7 Membership	H8 Membership	Primary Path
Shandong	0.38	0.82	0.42	0.78	0.35	0.42	0.72	0.45	H2/H4/H7
Henan	0.22	0.20	0.30	0.68	0.28	0.32	0.38	0.65	H4/H8
Hubei	0.35	0.30	0.72	0.40	0.45	0.68	0.38	0.42	H3/H6
Hunan	0.25	0.22	0.38	0.35	0.58	0.40	0.28	0.35	H5
Guangdong	0.96	0.40	0.42	0.45	0.32	0.42	0.38	0.36	H1
Guangxi	0.15	0.16	0.18	0.20	0.16	0.18	0.16	0.18	—
Hainan	0.12	0.14	0.14	0.15	0.12	0.14	0.14	0.14	—
Chongqing	0.25	0.28	0.32	0.35	0.28	0.30	0.32	0.30	—
Sichuan	0.22	0.24	0.35	0.58	0.30	0.35	0.55	0.32	H4/H7
Guizhou	0.12	0.14	0.14	0.16	0.12	0.14	0.14	0.14	—
Yunnan	0.14	0.16	0.16	0.18	0.14	0.16	0.16	0.16	—
Tibet	0.08	0.10	0.10	0.10	0.08	0.10	0.10	0.10	—
Shaanxi	0.18	0.20	0.24	0.26	0.20	0.22	0.22	0.22	—
Gansu	0.10	0.12	0.12	0.14	0.10	0.12	0.12	0.12	—
Qinghai	0.10	0.10	0.10	0.12	0.10	0.10	0.10	0.10	—
Ningxia	0.12	0.14	0.14	0.16	0.12	0.14	0.14	0.14	—

Province	H1 Membership	H2 Membership	H3 Membership	H4 Membership	H5 Membership	H6 Membership	H7 Membership	H8 Membership	Primary Path
Xinjiang	0.14	0.16	0.16	0.18	0.14	0.16	0.16	0.16	—

Note: The path numbers correspond one-to-one with H1–H8 in Table 4-2.

Appendix C-2 Attribution Rationale for Representative Provinces by Path

Path	RepresentativeProvinces	AttributionRationale
H1（SC·AA·HE·GC·SI·SE）	Beijing, Shanghai, Jiangsu, Zhejiang, Guangdong	All six core conditions are well above average, with outstanding performance in the number of sports social organizations, use of systemic reform instruments, per capita sports venue area, policy supply, and instrument utilization.
H2（GC·SE）	Shandong	Government service capacity and social sports instructor workforce are prominent, with clear policy safeguards and working mechanisms; the national fitness public service system continues to improve.
H3（PP）	Hubei, Fujian	Policy supply intensity is prominent; Hubei implements the "Double Innovation" initiative, and Fujian has included national fitness facility construction in its practical projects for the people for 23 consecutive years.
H4（SC）	Hebei, Jiangsu, Zhejiang, Shandong, Henan, Sichuan	Robust venue and facility construction, with per capita sports venue area generally at or above the national average; Jiangsu ranks first nationally, and Sichuan has achieved the highest cumulative growth nationwide.
H5（PP·AA·SI）	Anhui, Hunan	Policy supply and institutional setup advance synergistically; Anhui implements the "Happy Fitness Action," and Hunan has achieved notable growth in per capita sports venue area.
H6（SC·HE·SI·SE）	Fujian, Hubei	Synergistic combination of venue facilities, health education, and human resources; Hubei has won awards in social sports instructor exchange exhibitions, and Fujian has established a national physique monitoring data platform.
H7（SC·GC）	Shandong, Sichuan	Dual drivers of venue facilities and government service capacity; Shandong has fully opened public sports venues and facilities to the public, and Sichuan is committed to building a higher-level national fitness public service system.
H8（SC·AA·SI·SE）	Henan	Collaborative effort of venue facilities, institutional setup, and human resources; administrative villages have achieved full coverage of sports facilities, and the sports sector has been included as a key provincial livelihood initiative.
